# Comparative assessment of airborne infection risk tools in enclosed spaces: Implications for disease control

**DOI:** 10.1016/j.idm.2024.11.003

**Published:** 2024-11-28

**Authors:** Amar Aganovic, Giorgio Buonanno, Guangyu Cao, Christian Delmaar, Jarek Kurnitski, Alex Mikszewski, Lidia Morawska, Lucie C. Vermeulen, Pawel Wargocki

**Affiliations:** a Department of Automation and Process Engineering, UiT the Arctic University of Norway, Tromsø, Norway; b Department of Civil and Mechanical Engineering, University of Cassino and Southern Lazio, Cassino, FR, Italy; c International Laboratory for Air Quality and Health, Queensland University of Technology, Brisbane, Qld, Australia; d Department of Energy and Process Engineering, Norwegian University of Science and Technology - NTNU, Trondheim, Norway; e Centre for Infectious Disease Control, National Institute for Public Health and the Environment (RIVM), Bilthoven, Netherlands; f Department of Civil Engineering and Architecture, Tallinn University of Technology, Tallinn, Estonia; g Department of Civil Engineering, Aalto University, Espoo, Finland; h Global Centre for Clean Air Research (GCARE), Department of Civil and Environmental Engineering, Faculty of Engineering and Physical Sciences, University of Surrey, Guildford, GU2 7XH, United Kingdom; i Department of Environmental and Resource Engineering, Technical University of Denmark, Copenhagen, Denmark

**Keywords:** Infection risk tools, Infection risk calculators, Airborne transmission, Respiratory virus

## Abstract

The COVID-19 pandemic, caused by SARS-CoV-2, highlighted the importance of understanding transmission modes and implementing effective mitigation strategies. Recognizing airborne transmission as a primary route has reshaped public health measures, emphasizing the need to optimize indoor environments to reduce risks. Numerous tools have emerged to assess airborne infection risks in enclosed spaces, providing valuable resources for public health authorities, researchers, and the general public.

However, comparing the outputs of these tools is challenging because of variations in assumptions, mathematical models, and data sources. We conducted a comprehensive review, comparing digital airborne infection risk calculators using standardized building-specific input parameters. These tools generally produce similar and consistent outputs with identical inputs. Variations mainly stem from model selection and the handling of unsteady viral load conditions. Differences in source term calculations, including particle emission concentrations and respiratory activity, also contribute to disparities. These differences are minor compared to the inherent uncertainties in risk assessment. Consistency in results increases with higher ventilation rates, showing a robust trend across models. However, inconsistencies arose in the inclusion of face masks, often due to the lack of detailed efficiency values. Despite some differences, the overall consistency underscores the value of these tools in public health strategy and infectious disease control.

We also compared some of the model's efforts to conduct retrospective assessments against reported transmission events by assuming input parameters to the models so that the calculated risk would closely fit the original outbreak infection rate. Thus, validating these models against past outbreaks remains challenging because of the lack of essential input information from observed events. This comparative analysis demonstrates the importance of transparent data sources and justifiable model assumptions to enhance the reliability and precision of risk assessments.

**Table:** Parameters used

Parameter	Symbol	Description
Air change rate	ACH	The number of times air is replaced in a room per hour
Aerosol diameter size	d	The diameter of aerosol particles (μm)
Biological decay rate	λ_decay_	The rate at which the virus decays biologically (h^−1^)
Deposition fraction	f_dep_(D)	The fraction of particles deposited in the respiratory tract (−)
Dose–response relationship	ID_63.2%_; ID_50%_	The infectious dose required to infect 63.2% or 50% of the population (RNA copies)
Droplet volume	DV	The volume of each aerosol/droplet (mL)
Event reproduction number	R	The number of new disease cases divided by the number of infectors
Efficiency of face mask	η	The inward efficiency of the face mask (values between 0 and 1)
Exhalation rate	ER	The volume of air exhaled per hour (m^3^/h) or per second (L/s)
Fraction of infectious virus	f_inf_	The fraction of airborne virus particles that remain infectious (−)
Gravitational settling	λ_dep_	The rate at which particles settle due to gravity (h^−1^)
Host immunity of the exposed host	HI_exp_	The immunity level of the exposed individual (−)
Infection risk probability	P	The probability of infection (%)
Inhalation rate	IR	The volume of air inhaled per hour (m^3^/h)
Number of infectious individuals	I	The number of infectious individuals in the room
Number of susceptible individuals	N_s_	The number of susceptible individuals in the room
Particle emission concentration	PEC	The number of particles emitted per cubic centimeter (#/cm^3^)
Particle emission flow	PEF	The flow of emitted particles per second (#/s)
Probability of infection (event-based)	P_event_	Probability of infection based on reproduction number and population size (−)
Quanta concentration in air	n(t)	Quanta concentration in the air over time (quanta/m^3^)
Quanta emission rate	S	The rate of quanta emission per hour (quanta/h)
Relative percentage difference	RPD	The relative percentage difference between two tools (%)
Relative reduction index	$\Delta P_{rel.}$	The relative reduction in infection risk (%)
Removal mechanisms	λ_1_, λ_2_	The rate of removal mechanisms before and after intervention (h^−1^)
Room volume	V_room_	The volume of the room (m^3^)
Total exposure time	t	The total time of exposure (hours)
Transmissibility factor	T_voc_	Transmissibility factor for the variant of concern (−)
Ventilation rate	λ_dep_	The rate at which air is ventilated (h^−1^)
Viral concentration in air	N(t)	Viral concentration in the air over time (RNA/m^3^)
Viral load	VL	The concentration of viral RNA copies per milliliter (RNA/mL)

## 1 Introduction

Infectious respiratory diseases have been causing significant public health challenges worldwide, with outbreaks and pandemics threatening populations and straining healthcare systems. The latest SARS-CoV-2 pandemic, which emerged in late 2019, became one of the most significant global health crises of modern times. According to some estimates of excess deaths compared to previous pandemics, COVID-19 is the largest (0.15–0.28%) since the 1918–20 H1N1 influenza pandemic (1%) when scaled to 2020 populations (Simonsen & Viboud, 2021).

It took immense pressure at the beginning of the pandemic to make the World Health Organization (WHO) acknowledge the importance of airborne transmission at all. Almost 2 years after the start of the pandemic, backed by strong support from research evidence, the 10.13039/100004423WHO finally recognized that the primary mode of COVID-19 transmission is through airborne transmission (Lewis, 2022). The highly contagious nature of SARS-CoV-2 and its ability to transmit by air has made containment and control efforts extremely complex. Mitigation strategies against airborne transmission in indoor environments have included prevention measures such as social distancing and later wearing face masks (Ayouni et al., 2021), and strategies to improve the indoor air quality with enhanced ventilation, air purifiers, and/or filtration of recirculated air. Thus, the formal recognition of the importance of airborne transmission has significant implications for the recommendation of disease control measures, as it requires the risk of transmission to be controlled beyond maintaining physical distance and wearing masks in close-contact settings.

Predicting the airborne transmission risk in enclosed spaces is becoming an essential part of any occupational health and safety risk assessment. During the pandemic, many open-access digital tools emerged to assess airborne transmission risk, providing real-time data and facilitating evidence-based decision-making for public health authorities, researchers, and the general public (Albettar et al., 2022; National Geographic, 2020). To generate exposure scenarios and infection risk estimates, users have the ability to select and/or adjust a wide range of input parameters, including occupancy levels, viral load, exposure time, floor area or room size, outdoor air ventilation, recirculation rates, duct filter types, use of air cleaners and their capacities, and mask types (Aganovic et al., 2023a; Harmon & Lau, 2022a). These web-based tools can play a pivotal role in helping identify optimal strategies for infectious disease control in enclosed spaces by public health authorities. In the post-pandemic future, there will be an increasing need for user-friendly tools to consider the appropriate mitigation and risk control measures for indoor spaces (Choi et al., 2016).

However, several potential issues may arise when comparing the outputs of different tools calculating indoor airborne transmission risk. Firstly, different tools might use varying assumptions and input parameters, with discrepancies in these parameters potentially leading to significantly different risk assessments. Secondly, the underlying mathematical models and algorithms employed by these tools can differ in complexity and accuracy. Although most of these tools are based on relatively simple exponential dose–response models with exposure concentrations based on a completely mixed room assumption (Guo et al., 2021), others may incorporate more parameters. As a result, the outputs may vary significantly in terms of reliability and precision (Sze et al., 2010). Thirdly, the quality of data used to develop these tools plays a vital role in their accuracy. Tools relying on data from different sources for important inputs such as infectious dose or mask efficiency, or using outdated information, may produce inconsistent results (Aganovic & Kadric, 2023). Thus, comparing multiple tools and cross-referencing their outputs may not only help improve the reliability of risk assessments but also provide a comprehensive insight for developing a much-needed standardized approach for these tools. At present, no such comparison is available. We conducted a search strategy to select and compare the outputs of digital airborne infection risk calculators by employing the same building-specific input parameters/values. To grasp any potential differences in outputs, we solely considered studies that reported/described the mathematical modeling approach employed in the development of these tools. Specifically, the study aimed to.

1. Investigate the discrepancies in output results among different tools available on the web used for airborne infection risk assessment during the COVID-19 pandemic.
2. Explore the underlying factors contributing to variations in these output results, including differences in assumptions and mathematical models, and data sources.

By achieving these objectives, the study aimed to provide insights into the strengths and limitations of existing tools, ultimately contributing to the enhancement of infectious disease control measures in enclosed spaces and offering guidance for the development of standardized approaches for such tools in the future.

## 2 Methodology: selecting tools for comparison assessment

We conducted a scoping review using the following search engines: PubMed, SpringerLink, ScienceDirect, and Google Scholar. The duration of the search was from 2019 to 2023. The search keywords were combined using Primary Medical Subject Headings (MeSH) and Boolean terms. The main keywords used included “airborne risk assessment”, “airborne infection risk calculator” and “indoor infection risk calculator”.

Articles were included if they met the following criteria.

• The authors introduced a novel risk calculator designed to assess airborne infection risk, or they verified or made enhancements to an existing calculator for airborne infection risk assessment
• The article provided a clear description of the mathematical modeling approach that underlies the predictive capability of the calculator.
• The risk calculator is available as an interactive open-access web-based application tool.
• The search was limited to English-language web-based online tools.

Our search resulted in 676 articles after excluding duplicates. Of these, 46 articles passed the title and abstract stage, and the complete text was evaluated. A total of 12 articles met the eligibility criteria. Table forms were developed to record basic descriptive data for each calculator, including the name of the proposed or analyzed model, the type of the output infection risk equation, and the methodology for the development (or analysis) of each model's input parameters. A detailed overview of the calculators is presented below in Table 1, Table 2. We only compared the airborne infection calculators where input on the viral load or quanta emission rate was provided, as otherwise, relative comparisons would not be relevant. This included eight studies for viral-based load input (Aganovic et al., 2023b; Azimi et al., 2021; de Oliveira et al., 2021; Lau et al., 2022; Lelieveld et al., 2020; Parhizkar et al., 2022; Schijven et al., 2021; World Health Organization, 2024) and four studies for quanta emission-based load input (Harmon & Lau, 2022b; Kurnitski et al., 2023; Mikszewski et al., 2021; Peng et al., 2022). Because the outcome infection risk did not respond to changing input parameters, AIRVICA (Lau et al., 2022) software was excluded from the viral-based load studies. The Facility Infection Risk Estimator™ (Harmon & Lau, 2022b) was excluded because it was not possible to control the exposure time. Covid-19 Risk Calculator (Azimi et al., 2021) and Safe Air Spaces (Parhizkar et al., 2022) were excluded because neither calculator provided a viral load input.

**Table 1:** Descriptive overview of infection risk calculators (Part I).

Name of web-tool	Virus variant	Infectious dose source		Dose-response relationship	Particle concentration (#/cm^3^)	Exhalation rate	Expiratory/physical activity mode	Aerosol size distribution mode
		Quanta	Viral load					
Airborne.cam (de Oliveira et al., 2021)	N/A	Viral load based	3 viral loads	N/A	N/A	N/A	3 modes	5 cut-off diameter sizes
AIRVICA (Lau et al., 2022)	N/A	Viral load based	Custom	Custom	Custom	Custom	3 modes	N/A
Airborne Infection Risk Calculator (Mikszewski et al., 2021)	N/A	N/A	Quanta based	N/A	N/A	Custom	15 modes	N/A
AirCoV2 (Schijven et al., 2021)	N/A	Viral load based	10 viral loads	Custom	N/A	N/A	9 modes	N/A
ARIA (World Health Organization, 2024)	6 variants	Viral load based	7 viral loads	N/A	N/A	N/A	Custom	N/A
COVID 19 Aerosol Transmission Risk Calculator (Lelieveld et al., 2020)	N/A	Viral load based	Custom value between 10^8 and 10^11 RNA/ml	Custom dose (TRCID_50_) between 100 and 1000 RNA copies	N/A	Custom	3 modes	Custom choice of mean diameter
COVID-19 Aerosol Transmission Estimator (Peng et al., 2022)	N/A	Custom value	Quanta based	N/A	N/A	Custom	Custom	N/A
COVID-19 Risk Calculator (Azimi et al., 2021)	N/A	Viral load based	N/A	N/A	N/A	Custom	4 modes	N/A
Facility Infection Risk Estimator™ (Harmon & Lau, 2022b)	N/A	3 values per activity mode	Quanta based	N/A	N/A	N/A	5 modes	N/A
New Dose-Response Model (Aganovic et al., 2023b)	4 variants	Viral load based	Custom	N/A	N/A	N/A	3 modes	N/A
REHVA Calculator (Kurnitski et al., 2023)	N/A	Custom value	Quanta based	N/A	N/A	Custom	N/A	N/A
Safe Air Spaces (Parhizkar et al., 2022)	N/A	Viral load based	N/A	N/A	N/A	N/A	N/A	N/A

**Table 2:** Descriptive overview of infection risk calculators (Part II).

Name of web-tool	Room characteristics			Exposure time	Occupancy rate		Removal mechanisms						
	Volume	Area	Height		No. of infected	No. of susceptible	Ventilation	Filter	Gravitational settling	Biological decay	UV	Air Cleaner	Mask removal efficiency
Airborne.cam (de Oliveira et al., 2021)	Custom	Custom	Custom	Custom	Custom	Custom	Custom	5 types	N/A	N/A	N/A	N/A	4 types
AIRVICA (Lau et al., 2022)	Custom	Custom	Custom	Custom	Custom	Custom	Custom	N/A	Custom	Custom	N/A	N/A	Custom
Airborne Infection Risk Calculator (Mikszewski et al., 2021)	Custom	Custom	Custom	Custom	Custom	Custom	Custom	N/A	Custom	Custom	N/A	N/A	N/A
AirCoV2 (Schijven et al., 2021)	Custom	Custom	Custom	Custom	Fixed (1 infected person)	Custom	Custom	N/A	N/A	N/A	N/A	N/A	N/A
ARIA (World Health Organization, 2024)	Custom	Custom	Custom	Custom	Custom	Custom	Custom	Custom	N/A	N/A	Custom	Custom	3 types
COVID 19 Aerosol Transmission Risk Calculator (Lelieveld et al., 2020)	Custom	Custom	Custom	Custom	Custom	Custom	Custom	N/A	N/A	N/A	N/A	Custom	Custom
COVID-19 Aerosol Transmission Estimator (Peng et al., 2022)	Custom	Custom	Custom	Custom	Custom	Custom	Custom	Custom	Custom	Custom	N/A	N/A	Custom
COVID-19 Risk Calculator (Azimi et al., 2021)	Custom	Custom	Custom	Custom	Custom	N/A	Custom	Custom	N/A	N/A	Custom	Custom	Custom
Facility Infection Risk Estimator™ (Harmon & Lau, 2022b)	Custom	Custom	Custom	N/A	Custom	Custom	Custom	8 types	N/A	9 values based on relative humidity	Custom	Custom	13 types
New Dose-Response model (Aganovic et al., 2023b)	Custom	N/A	N/A	Custom	Custom	Custom	Custom	Custom	N/A	4 values based on relative humidity	3 values	N/A	3 types
REHVA Calculator (Kurnitski et al., 2023)	Custom	Custom	Custom	Custom	Custom	Custom	Custom	N/A	Custom	N/A	Custom	Custom	Custom
Safe Air Spaces (Parhizkar et al., 2022)	Custom	Custom	Custom	Custom	Custom	N/A	Custom	N/A	N/A	N/A	N/A	N/A	20 types

We conducted both absolute infection risk and a relative comparative assessment using the relative reduction index $(\Delta P_{rel.}$) and the relative percent difference (RPD). The relative reduction in infection risk is defined as:

(1) $$\Delta P_{rel.}=100\%\cdot \frac{P_{0,5\;ACH\;}-P_{5.0\;ACH\;}}{P_{0,5\;ACH\;}}\;\left(\%\right)$$

when increasing the ventilation rate from 0.5 to 5.0 air changes per hour (ACH) for ventilation while wearing a surgical mask:

(2) $$\Delta P_{rel.}=100\%\cdot \frac{P_{no\;mask\;}-P_{surgical\;mask\;}}{P_{no\;mask}}\;\left(\%\right)$$

(3) $$\text{The}\;\text{RPD}\;\text{between}\;\text{tools}\;1\;\text{and}\;2\;\text{is}\;\text{defined}\;\text{as}\;RPD=100\%\cdot 2\cdot \frac{P_{tool1\;}-P_{tool2\;}}{P_{tool1}+P_{tool2}}$$

## 3 Results

The airborne infection risk tools do not provide options to modify or select any removal mechanisms or preventive measures, except for ventilation, air cleaners (only available in two tools), and the use of face masks (offered by several tools for both viral-based and quanta-based methods). As an air cleaner essentially delivers clean air similarly to ventilation, it was deemed unnecessary to compare this removal mechanism. The other viral-based tools did not offer the option to change either the decay or the gravitational settling values. Therefore, we opted only to compare the tools for different ventilation rates and face masks. The reason for treating quanta-based models separately is that the papers on quanta-based models do not provide data on the calculation of volume emission rate, viral load, and the quanta-response relationship. This lack of data makes it impossible to back-calculate the viral load and compare it against viral-based models.

### 3.1 Viral-based load calculator tools

The comparison was made for a typical classroom with an area of 56.2 m^2^ and height of 3.0 m. The classroom contained one infected person who was constantly speaking and 25 susceptible individuals. Three input viral loads were used for the infected person, namely 10^8^, 10^9^, and 10^10^ RNA copies/milliliter (mL), while two ventilation air exchange rates were compared, 0.5 and 5.0 ACH. The total exposure time considered was 1 h. In addition to ventilation, we also compared the impact of face masking on the tools' infection risk output. Because only two tools (REHVA and COVID-19 Aerosol Transmission Estimator) provided the option to change face mask efficiency for quanta-based tools, and the infection risk output of one of these tools (COVID-19 Aerosol Transmission Estimator) did not respond to changes in mask efficiency, we decided to omit face mask comparison for quanta-based tools. To understand any possible output differences between the respective risk calculators, the complete model descriptions including input parameters are presented in Table 3, Table 4.

**Table 3:** Infection risk calculation equation models.

Infection risk tool	Infection risk model: Probability of infection (−)
**Airborne.cam** (de Oliveira et al., 2021)	$1-e^{-\frac{IR\cdot \int_{0}^{t}N\left(t\right)dt}{{ID}_{63.2\%}}}$
**AirCoV2** (Schijven et al., 2021)	$1-e^{-\frac{IR\cdot Pois\left(\int_{0}^{t}N\left(t\right)dt\right)}{{ID}_{63.2\%}}}$
**ARIA** (World Health Organization, 2024)	$1-e^{-\ln\;2\cdot T_{voc}\cdot \left(\frac{1}{1-HI_{\exp}}\right)\frac{\int_{0}^{D_{\max}}\left(\int_{t_{1}}^{t_{2}}N\left(t,D\right)dt\cdot f_{\inf}\cdot IR\cdot f_{dep}\left(D\right)\cdot \left(1-\eta_{\inf}\right)\right)dD}{{ID}_{50\%}}}$
**COVID 19 Aerosol Transmission Risk Calculator** (Lelieveld et al., 2020)	$1-{\left(1-0.5\right)}^{\frac{IR\cdot N\cdot t}{{ID}_{50\%}}}$
**New Dose Response Model** (Aganovic et al., 2023b)	$1-e^{-\frac{IR\cdot \int_{0}^{t}N\left(t\right)dt}{{ID}_{63.2\%}}}$

**Table 4:** Model and input parameter descriptions for viral-load-based infection calculators.

Web-based tool	Airborne.cam (Mikszewski et al., 2021)	AirCoV2 (Schijven et al., 2021)	ARIA (World Health Organization, 2024)	COVID-19 Aerosol Transmission Risk Calculator (Peng et al., 2022)	New Dose Response Model (Aganovic et al., 2023b)
**Viral concentration in air**N**(RNA/m**^**3**^**)**	Solved for N by first order balance model: $V_{room}\cdot \frac{dN\left(t\right)}{dt}=n\cdot S-V_{room}\cdot N\left(t\right)\cdot \sum \lambda$				
**Steady-state conditions**	$\frac{dN}{dt}\neq 0$			$\frac{dN}{dt}=0$	$\frac{dN}{dt}\neq 0$
**Inhalation rate**IR**(m**^**3**^**/h)**	1.87 m^3^/h	Normally distributed on log-scale with mean = 0.41 l m^3^/h and SD = 0.003 m^3^/h	Predetermined values based on age and physical activity (U.S. Environmental Protection Agency, 2011)	0.60 m^3^/h	0.52 m^3^/h
**Dose–response relationship**	ID_63.2%_ = 410 RNA copies	ID_63.2%_ = 1440 RNA copies	Calculated (Schijven et al., 2021)	ID_50%_ = 316 RNA copies	ID_63.2%_ = 14 000 RNA copies
**Viral emission rate**S**(RNA/h)**	PEC·ER·DV·VL				PEF·VL·DV
**Particle emission flow**PEF**(#/s)**	N/A				Extracted from Fleischer et al. (Fleischer et al., 2022)
**Particle emission concentration PEC (#/cm**^**3**^**)**	B-L-O method (Alsved et al., 2020)	Calculated based on data from seven studies (Alsved et al., 2020; Asadi et al., 2019; Duguid, 1946; Fabian et al., 2011; Gerone et al., 1966; Lindsley et al., 2012; Mürbe et al., 2020)	B-L-O method (Johnson et al., 2011)	0.06 #/cm^3^ (breathing) 0.6 #/cm^3^ (speaking) 6 #/cm^3^ (singing)	N/A
**Droplet volume DV (mL)**	Not described		Each aerosol/droplet from different diameter size bins is calculated as the volume of a perfect sphere		
**Exhalation rate ER (m**^**3**^**/h) or (l/s)**	0.75 m^3^/h (speaking) 4.50 m^3^/h (coughing)		Predetermined values based on age and physical activity (U.S. Environmental Protection Agency, 2011)	0.60 m^3^/h	0.52 m^3^/h
**Aerosol diameter *d* size distribution (**μm)	5 aerosol cut classes from 5 μ m to 100 μ m	6 aerosol droplet size classes from 0.3 μ m to >10 μ m	0.8 μm ≤ d ≤ 1000 μm	d ≤ 10 μm	d ≤ 10 μm
**Gravitational settling (h^−1^)**	0.39 h^−1^	0.00 h^−1^	0.054 h^−1^ (breathing) 0.146 h^−1^ (speaking) 0.167 h^−1^ (shouting)	0.00 h^−1^	Calculated based on Stokes' law for each diameter size bin
**Biological decay rate (h^−1^)**	0.63 h^−1^	0.48 h^−1^	0.63 h^−1^ (RH > 40%) 0.11 h^−1^ (RH < 40%)	0.59 h^−1^	0.48 h^−1^ (RH = 53%) 1.050 h^−1^ (RH = 70%) 2.4 h^−1^ (RH = 81%)
**Ventilation rate (h^−1^)**	Custom (see case scenarios)				

#### 3.1.1 Ventilation

**Fig. 1:**
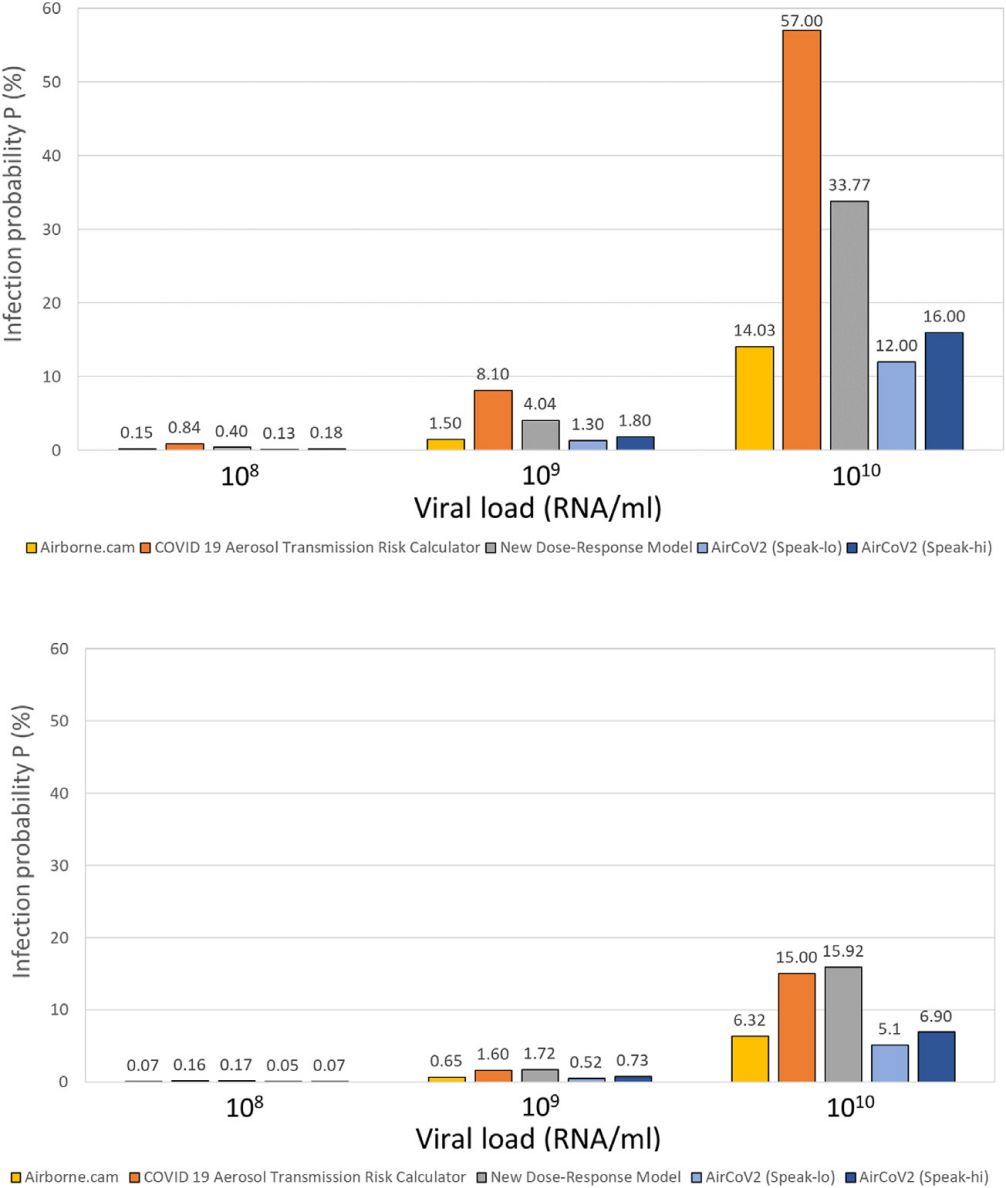
Infection risk probability after 1 h of exposure time in a classroom ventilated at 0.5 air changes per hour (ACH) (upper) and 5.0 ACH (lower).

Fig. 1 depicts the absolute comparison of the viral-load-based risk calculators for typical classroom settings at 0.5 and 5 ACH for the New Dose Response Model, Airborne.cam, and COVID-19 Aerosol Transmission Risk Calculator given the three included viral loads in Airborne.cam (10^8^, 10^9^, and 10^10^ RNA copies/mL). Fig. 1 shows that the three infection risk calculators yield different absolute infection risk values, despite employing the same input parameters that can be controlled across all three tools—viral load, air exchange rate, respiratory activity, and exposure time.

The infection outputs generated by the COVID-19 Aerosol Transmission Risk Calculator and the New Dose Response Model exhibit greater similarity to each other when compared to Airborne.cam. This observation may be attributed to several factors. Firstly, in the exponential models, the computation of airborne viral concentration takes place under unsteady conditions (dN/dt ≠ 0), involving a solution to a first-order differential equation. Conversely, the COVID-19 Aerosol Transmission Risk Calculator assumes steady-state conditions and resolves the viral mass balance model through a simple linear equation. Consequently, even if all input parameters were identical, the results would diverge because of variations in the time required to reach a steady-state viral concentration within a well-mixed volume of room air. Therefore, the difference between the COVID-19 Aerosol Transmission Risk Calculator and the other models seems (as dose–response is not so different) to stem mostly from the steady-state assumption. It is easy to see that in the low ventilation scenario, the air concentration is well away from steady state after 1 h, leading to a significant overestimation by the COVID-19 Aerosol Transmission Risk Calculator, whereas for ACH = 5, the steady state is a much better approximation and the COVID-19 Aerosol Transmission Risk Calculator projection pulls closer to the other tools. This finding thus seems somewhat situational; it comes from the specific scenario assumptions and is not a general finding.

It should be noted here that the dose–response relationships for Airborne.cam and COVID-19 Aerosol Transmission Risk Calculator are based on data for SARS-CoV-1, while the New Dose Response Model and AirCoV2 use more recent data extracted from SARS-CoV-2 studies. Furthermore, these web tools adopt different approaches in handling the source term within the mass balance equation. Specifically, all the infection risk tools except for the New Dose Response Model use a particle-emission concentration derived from experimental studies to calculate the exhaled quantity of viral load. In contrast, the New Dose Response Model uses a source term calculation approach based on particle emission flow (#/s) from experimental studies. Among the other source parameters that remain constant, the models employ distinct input values, except for the controlled viral load. Notably, this variation extends to parameters such as the droplet volume emission rate and exhalation rate, which cannot be adjusted, except in the case of the Airborne.cam model. Lastly, the removal mechanisms differ among the models. Neither AirCoV2 nor COVID-19 Aerosol Transmission Risk Calculator incorporate gravitational settling into their calculations. While there are variations in inactivation rates due to biological decay, these differences typically fall within a relatively narrow range of 0.4–0.6 h^−1^, depending on the reference source. As depicted in Fig. 1, it is evident that higher ventilation removal rates have a more pronounced impact in the logarithmic-based model of the COVID-19 Aerosol Transmission Risk Calculator, in contrast to the exponential-based models used in the other two tools.

It is noteworthy that the differences in the dose–response parameters for the viral-load models (Table 4) are substantial. However, this variation does not appear to be reflected in the quantitative comparison results, where the differences in model projections are relatively modest. This discrepancy warrants further discussion. One plausible explanation is that the variability in the dose–response parameters may be mitigated by other factors in the models, such as differences in viral emission rates, inhalation rates, or removal mechanisms.

**Fig. 2:**
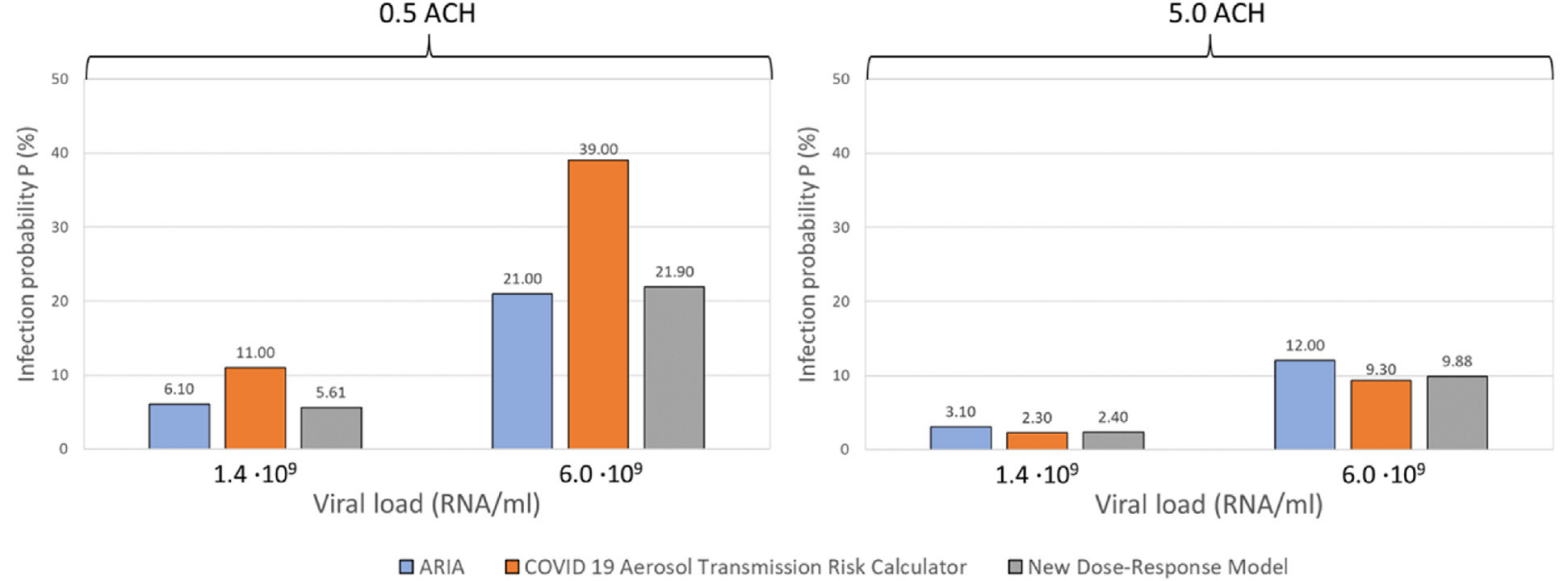
Infection risk probability after 1 h of exposure time in a classroom ventilated at 0.5 air changes per hour (ACH) (left) and 5.0 ACH (right).

We further compared the New Dose Response Model and COVID-19 Aerosol Transmission Risk Calculator against ARIA based on two different viral loads, $1.4\cdot 10^{9}$ and, $6.0\cdot 10^{10}$ RNA/mL (as available in ARIA) as shown in Fig. 2.

The relative percentage difference (RPD) metric calculated by Airborne.cam shows results most similar to those of AirCoV2, whereas ARIA yields results more closely aligned with the New Dose Response Model, followed by the COVID-19 Aerosol Transmission Risk Calculator. As indicated in Table 5, the RPD metric is influenced by the viral load (RNA/mL).

**Table 5:** Relative percentage difference between viral-load-based infection risk calculators when comparing different ventilation rates.

ACH	RNA/mL	Airborne.cam				ARIA		COVID 19 ATRC			N-DRM	
		COVID 19 ATRC	N-DRM	AirCoV2 (lo)	AirCoV2 (hi)	COVID 19 ATRC	N-DRM	N-DRM	AirCoV2 (lo)	AirCoV2 (hi)	AirCoV2 (lo)	AirCoV2 (hi)
0.5	10^8^	139%	91%	14%	18%	–	–	73%	146%	129%	102%	76%
	10^9^	138%	92%	14%	18%	–	–	67%	145%	127%	103%	77%
	1.4 · 10^9^	–	–	–	–	57%	8%	65%	–	–	–	–
	6.0 · 10^9^	–	–	–	–	60%	4%	56%	–	–	–	–
	10^10^	121%	83%	16%	13%	–	–	51%	130%	112%	95%	71%
5	10^8^	78%	83%	33%	0%	–	–	6%	109%	83%	109%	109%
	10^9^	84%	90%	22%	12%	–	–	7%	102%	52%	107%	81%
	1.4 · 10^9^	–	–	–	–	30%	25%	4%	–	–	–	–
	6.0 · 10^9^	–	–	–	–	25%	19%	6%	–	–	–	–
	10^10^	81%	86%	21%	9%	–	–	6%	99%	74%	103%	79%

#### 3.1.2 Face masks

**Fig. 3:**
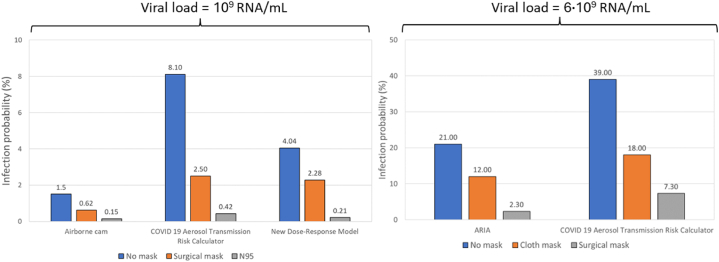
Probability of infection risk after 1 h of exposure in a classroom ventilated at 0.5 air changes per hour (ACH) for different types of face masks.

Fig. 3 illustrates the infection probability after 1 h of exposure in a classroom ventilated at 0.5 ACH for various face mask types, as calculated by different airborne infection risk calculators: Airborne.cam, COVID-19 Aerosol Transmission Risk Calculator, New Dose Response Model, and ARIA.

In this case, the calculators on the left part of the figure represent scenarios where only the infected person was wearing a face mask, while the right part depicts scenarios where all individuals in the room, both infected and exposed, were wearing masks. The differences in infection risk outputs between different calculators are caused by variations in their mathematical models as described in Section 3.1.1 and detailed in Table 4. These variations in modeling approaches, including the use of different dose–response relationships and assumptions about steady-state versus unsteady conditions, lead to absolute differences in the calculated infection probabilities.

The RPD metric in Table 6 compares the infection risk outputs between various airborne infection risk calculators, focusing on different mask-wearing scenarios. Similarly to ventilation rates, the RPD metric is influenced by the viral load (RNA/mL). However, as shown in Table 6, there is no consistent trend indicating whether the RPD increases or decreases with higher mask efficiency.

**Table 6:** Relative percentage difference between viral-load-based infection risk calculators when comparing different types of face masks.

RNA/mL	Face mask	Airborne.cam		COVID 19 ATRC	ARIA
		COVID-19 ATRC	New-dose response model	New-dose response model	COVID-19 ATRC
10^9^	No mask	138%	92%	67%	–
	Surgical mask	121%	114%	9%	–
	N95	95%	33%	67%	–
6.0 · 10^9^	No mask	–	–	–	60.00%
	Cloth mask	–	–	–	40.00%
	Surgical mask	–	–	–	104.17%

### 3.2 Quanta-based load calculator tools

To compare the three quanta-emission-based web tools, we used an identical classroom case scenario, differing only in the use of quanta/h values of 3.8, 38, and 380 instead of viral-based loads. It is important to note that compared to the REHVA calculator and the COVID-19 Aerosol Transmission Estimator, the user in AIRC defines the metabolic and respiratory activities, and the tool determines the corresponding quanta. This is an important difference in the approach, as it allows for a more customized and precise assessment of the aerosol transmission risk based on the specific activities and conditions defined by the user.

Once again, to comprehend any potential disparities in output, we present the full model description, including input parameters, in Table 7 for the respective risk calculators.

**Table 7:** Model and input parameter descriptions for quanta-load-based infection calculators.

Web-based tool	REHVA Calculator	COVID-19 Aerosol Transmission Estimator	AIRC
Infection risk model: *P* (%)	$\left(1-e^{-IR\cdot \int_{0}^{t}n\left(t\right)dt}\right)\cdot 100$		
Quanta concentration in air n (quanta/m^3^)	Solved for n by first-order balance model $V_{room}\cdot \frac{dn\left(t\right)}{dt}=S-V_{room}\cdot n\left(t\right)\cdot \sum \lambda$ ; $\left(\frac{dN}{dt}\neq 0\right)$		
Inhalation rate IR (m^3^/h)	0.65 m^3^/h	0.52 m^3^/h	0.54 m^3^/h
Quanta emission rate S (quanta/h)	Custom (see case scenarios)		
Gravitational settling ($h^{-1}$)	0.24 $h^{-1}$	0.24 $h^{-1}$	0.24 $h^{-1}$
Biological decay rate ($h^{-1}$)	0.63 $h^{-1}$	0.62 $h^{-1}$	0.63 $h^{-1}$
Ventilation rate ($h^{-1}$)	Custom (see case scenarios)		

In comparison to the viral-based models, the quanta-based models necessitate less input data because of the simplification of the source term. As illustrated in Table 7 both web tools rely on identical exponential infection risk models and first-order differential balance models to calculate the quanta concentration in the air. For the sake of comparison, we kept the input parameters at their default values; otherwise, both models yield identical results.

#### 3.2.1 Ventilation

**Fig. 4:**
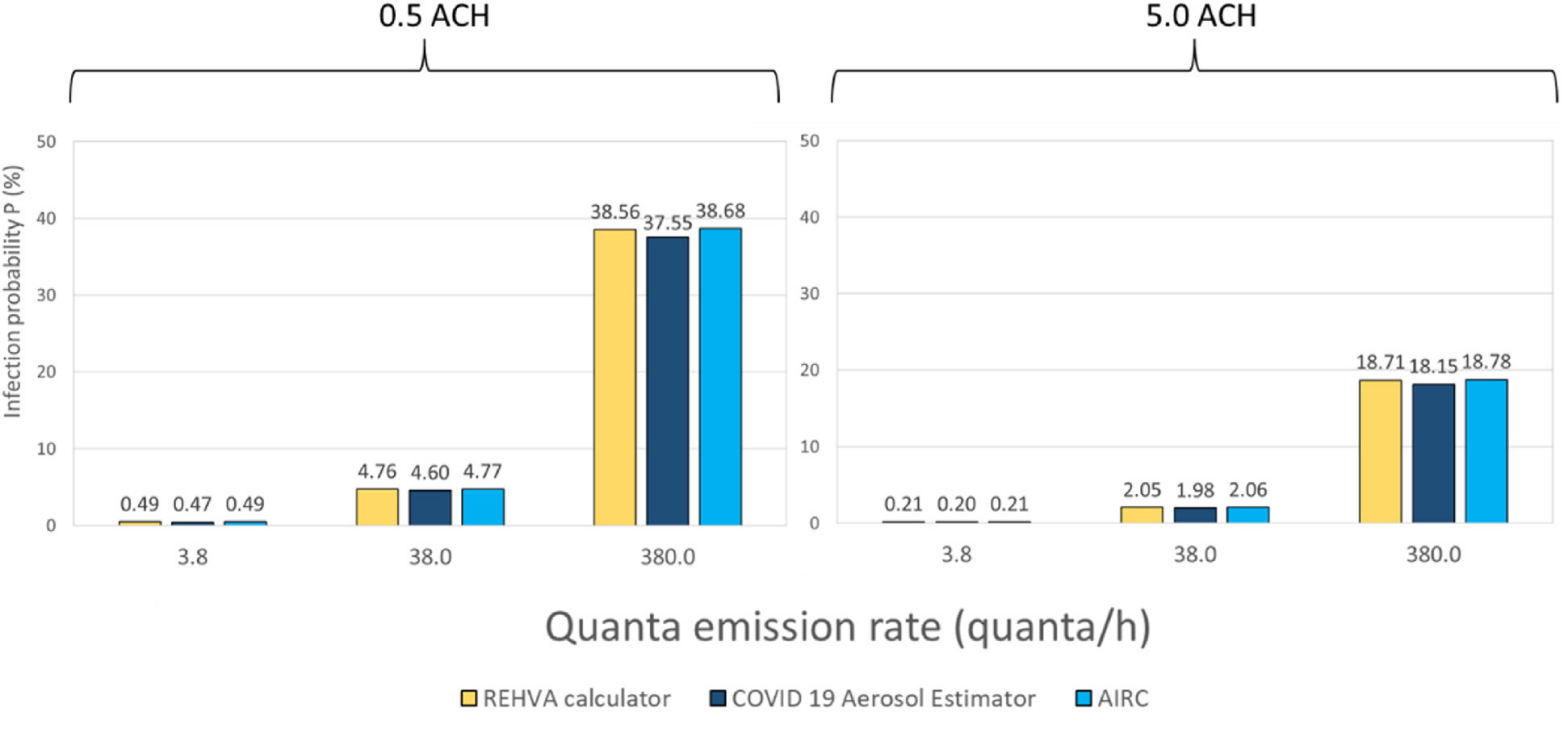
Infection risk probability after 1 h of exposure time in a classroom ventilated at 0.5 and 5.0 air changes per hour (ACH).

Fig. 4 depicts the infection risk when the room airflow rate is set to 0.5 ACH and 5.0 ACH. The slight differences observed in Fig. 4 can be easily explained by the differences in the input data, which were set to default values. Notably, the marginally higher inhalation rate and the lower gravitational deposition rate led to an increased infection risk output in the REHVA calculator. The infection output differences become lower when the ventilation rate is increased to 5 ACH.

In summary, the relative differences between the quanta-based tools are almost negligible compared to those of the viral-load-based tools, and these differences slightly decrease at higher ventilation rates as illustrated in Table 8.

**Table 8:** Relative percentage difference between quanta-based infection risk calculators.

ACH	Quanta/h	REHVA Calculator		COVID-19 Aerosol Estimator
		COVID-19 Aerosol Estimator	AIRC	AIRC
0.5	3.8	4%	0%	4%
	38	3%	0%	4%
	380	3%	0%	3%
5	3.8	5%	0%	5%
	38	3%	0%	4%
	380	3%	0%	3%

### 3.3 Relative reduction in infection risk by increasing ventilation rate and wearing surgical face masks

Increasing the ventilation rate by a factor of 10 leads to a reduction in infection risk of over 50%, regardless of the viral load, as illustrated in Table 9. This increase in ventilation can be considered quite substantial. As shown in Table 9, ARIA, Airborne.cam, AirCoV-2, and New Dose Response Model show similar results, within a 10% difference. Compared to the Airborne.cam model, the COVID-19 Aerosol Transmission Risk Calculator recorded at least 10% higher relative ventilation impact on infection risk compared to the other tools.

**Table 9:** Relative decrease in infection risk probability P(%) when the total ventilation rate is increased from 0.5 to 5.0 air changes per hour (ACH).

Viral Load (RNA/mL)	Tool					
	Airborne.cam	ARIA	COVID 19 ATRC	N-DRM	AirCoV2 (lo)	AirCoV2 (hi)
10^8^	53%	–	81%	58%	62%	61%
10^9^	57%		80%	57%	60%	59%
10^10^	55%		74%	53%	58%	57%
1.4 · 10^9^	–	49%	79%	48%	–	
6.0 · 10^9^		43%	76%	47%		

Quanta emission rate (quanta/h)	Tool		
	REHVA	COVID 19 AE	AIRC
3.8	57%	57%	57%
38	57%	57%	57%
380	51%	52%	53%

We note that the relative impact is dependent on the viral load for all five models. As in the case of viral-load-based web tools, increasing the ventilation rate by a factor of 10 results in a more than 50% reduction in infection risk, regardless of the quanta emission rate input. However, despite minor variations in absolute values, both the REHVA and COVID-19 Aerosol Transmission Estimator demonstrated identical results in terms of the relative impact of ventilation (Table 9).

As with ventilation, the relative impact of wearing a surgical face mask in viral-based models depends on the viral load (Table 10). For identical viral loads, all infection risk outputs differ by at least 10% when compared. One plausible explanation is that the infection risk models assume different surgical mask efficiencies.

**Table 10:** Percentage reduction in infection risk probability (P) when wearing a surgical face mask compared to not wearing one at 0.5 ACH.

Viral Load (RNA/mL)	Tool			
	Airborne.cam	ARIA	COVID 19 ATRC	N-DRM
10^9^	59%	–	69%	44%
6.0 · 10^9^	–	43%	54%	–

### 3.4 Relative risk reduction comparison with available hand calculation equations

Aganovic et al., 2024 (Aganovic et al., 2024) showed that the risk reduction in the steady state depends only on removal mechanisms before ($\sum \lambda_{1})$ and after ($\sum \lambda_{2})$ applying infection control measures:

(4) $$\Delta P_{abs.\max}=P_{1}-P_{2}=e^{-\frac{\sum \lambda_{1}\;\ln\frac{\sum \lambda_{2}}{\sum \lambda_{1}}}{\sum \lambda_{2}-\sum \lambda_{1}}}-e^{-\frac{\sum \lambda_{2}\;\ln\frac{\sum \lambda_{2}}{\lambda_{1}}}{\sum \lambda_{2}-\lambda_{1}}}$$

where $\Delta P_{abs.\max}$ is the maximum absolute infection risk difference (%) between two scenarios with removal mechanisms $\sum \lambda_{1}$ and $\sum \lambda_{2}$ (1/h). For this equation, the only input data needed is the air change rate of 0.5 and 5.0 ACH. With these values, equation (4) provides:

• *P*_*1*_ = 85.0%
• *P*_*2*_ = 35.8%
• $\Delta P_{abs.\max}$ = 49.2%
• $\Delta P_{rel.}$ = 57.9%

Thus, the result of the relative decrease of 58% is 1% higher compared to the typical values of 57% in Table 11. Another available equation is for a target ventilation rate developed by Kurnitski et al. (Kurnitski et al., 2023) for infection risk-based ventilation design:

(5) $$Q=q_{q}\left(N-1\right)-q_{r}V$$

**Table 11:** Probability of infection and relative reduction are calculated with equation (8) at specified quanta emission rates.

Quanta emission rate (quanta/h)	Infection probability *P* (%) at 0.5 ACH	Infection probability *P* (%) at 5.0 ACH	The relative decrease in infection risk probability $\Delta P_{rel.}$.(%)
3.8	0.94	0.22	76.7
38	9.38	2.19	76.7
380	93.8	21.9	76.7

To be used for the relative risk reduction calculation, *q*_*q*_ and *q*_*r*_ parameters are to be expanded:

(6) $$Q=\frac{qQ_{b}DN_{s}}{R}-\left(\lambda_{dep}+k\right)V$$

The probability of infection may be solved from the event reproduction number *R,* defined as the number of new disease cases divided by the number of infectors *R = N*_*c*_*/I.* As the number of new disease cases *N*_*c*_ = *p N*_*s*_ the individual probability can be calculated as follows:

(7) $$P=\frac{RI}{N_{s}}$$

where solving *R* from equation (6) and substituting to equation (8) provides for the probability of infection:

(8) $$P=\frac{qQ_{b}DI}{Q+\left(\lambda_{dep}+k\right)V}$$

Applying equation (7) with the same classroom input data, the probability of infection and the relative reduction can be calculated at specified quanta emission rates as shown in Table 11.

In this case, the infection probabilities at 5.0 ACH and 3.8 to 38 quanta/h values are close to the results in Fig. 4 thus showing good accuracy of this equation at low probability values which are of interest in most cases. Infection probabilities at 0.5 ACH are higher and also the relative decrease of 77% is higher in Table 11 compared to 57% in Table 9. These conservative values at higher infection probabilities reflect the accuracy decrease of the linearised dose–response model that has been used in the derivation of equation (4).

### 3.5 Reported use of the models for retrospective assessment of COVID-19 pandemic outbreaks

We found that four of the tools from Table 1 were retrospectively assessed against reported transmission events, namely the COVID-19 Aerosol Transmission Risk Calculator (Lelieveld et al., 2020), the COVID-19 Aerosol Transmission Estimator (Peng et al., 2022), the WHO ARIA tool (World Health Organization, 2024) and the Airborne Infection Risk Calculator (Mikszewski et al., 2021). Interestingly, all tools were retrospectively assessed for the same widely reported outbreak of SARS-CoV-2 at a choir rehearsal of the Skagit Valley Chorale (SVC) in March 2020 (Miller et al., 2021). After that rehearsal, 53 members of the SVC among 61 in attendance were confirmed or strongly suspected to have contracted COVID-19 and two died, yielding an infection risk of 87%. Using their tool, the COVID-19 Aerosol Transmission Risk Calculator, Lelieveld et al. (Lelieveld et al., 2020) predicted the high infection rate (>80%), while Peng et al. (Peng et al., 2022), using the COVID-19 Aerosol Transmission Estimator, predicted a secondary attack rate of 56/61 (92%). In other words, both tools claim that they match the Skagit infection numbers. Furthermore, the developers of the ARIA tool (World Health Organization, 2024) estimated that the secondary attack rate is between the 95th and 99th percentile of the infection risk from their model, which would translate to a viral load of the infected person between 1.4 and 6.0 $\cdot 10^{9}\;\frac{RNA}{mL}$. In contrast to the previous three applications, AIRC (Mikszewski et al., 2021) was used for a retrospective analysis applied to the SVC, determining that a quanta emission rate of 341 quanta h^−1^ is needed to reach an attack rate of 53% after 2.5 h of exposure. This emission rate occurs between the 92nd and 93rd percentile of the probability density function of quanta estimation rates (Buonanno et al., 2020) characteristic of an infected subject while singing. Although all three tools predicted high infection rates similar to the actual event, their predictions relied heavily on assumed input data to match the outbreak's infection rate closely. However, it is important to note that except for room volume and number of infected and susceptible individuals, input data of the values of the other numerous input parameters to the models were assumed so the calculated risk would closely fit the original outbreak infection rate. Numerous investigations in the context of the COVID-19 pandemic have neglected essential factors such as ventilation rates, space volume, filter and air cleaner efficiencies, and other building science features. The absence of this input data makes it challenging, if not impossible, to quantify the airborne risk linked to these conditions. Future reports on outbreaks must include this information to improve our understanding of the circumstances supporting airborne transmission of different diseases. It is also worth mentioning that retrospective assessments published to date were performed by authors who participated in developing these tools. In other words, we have only found one public health organization (WHO) (World Health Organization, 2024) that reports using these tools for either prospective or retrospective studies. The reasons for this may include:

i) Complexity and accessibility: Many tools are complex and require advanced technical expertise, making them inaccessible to the general public.
ii) Data availability and quality: As discussed, accurate assessment of airborne transmission risks relies heavily on real-time and high-quality data, including information about ventilation systems and the presence of infected individuals. In many real-life situations, obtaining such data is challenging, leading to unreliable results.
iii) Lack of awareness: Stakeholders, including healthcare professionals, policymakers, and the general public, are not aware of the existence and potential benefits of these tools. Raising awareness through education and outreach programs is essential.
iv) Trust and reliability: In some cases, skepticism regarding the accuracy and reliability of these tools may restrict their adoption. Ensuring transparent validation processes and providing evidence of their effectiveness can help build trust among potential users (as in point ii).
v) Policy and regulatory challenges: The absence of clear policies and regulations mandating the use of airborne transmission risk tools in specific settings may discourage their implementation. Collaborative efforts between researchers, policymakers, and regulatory bodies are necessary to address this issue.

## 4 Conclusion

In this study, we compared the outputs of digital airborne infection risk calculators using the same input parameters where applicable. We focused on studies that provided a clear description of the mathematical modeling approach underlying these tools and evaluated their quality based on input parameters related to viral characteristics, removal mechanisms, and respiratory characteristics.

Our comparison revealed that, despite some differences, the outputs of these tools showed a notable degree of similarity and consistency, even when using identical input parameters. In terms of model reliability and robustness, we recommend that viral-load-based calculators be favored over quanta-based models, particularly for specific case studies. This is because viral-load models provide greater flexibility in adjusting input parameters like inhalation rates and viral emission, offering a more tailored and accurate risk assessment. Quanta-based models, while useful, are more generalized and allow for fewer adjustments, making them less intuitive and potentially less accurate for public use. The choice of model and the handling of unsteady conditions in viral load calculations can lead to some variation in results. Furthermore, the methods for calculating source terms, including particle emission concentrations and their association with respiratory activity, vary among the tools, contributing to disparities in risk assessments.

However, these differences are relatively minor given the large uncertainties inherent in risk assessment. The similarities in the results across different tools underscore the robustness of these models in estimating airborne infection risks. Additionally, we observed that differences in outputs decrease with increasing ventilation rates, indicating a consistent trend across models.

We also compared the inclusion of different face masks in the models, and they did not show the same consistency in infection risk outputs with increasing mask efficiency. However, most models only provided the type of face mask and not the efficiency values, making it difficult to fully explain these inconsistencies.

This analysis underscores the need for transparent data sources and justifiable model assumptions to improve the reliability and precision of risk assessments. Despite some inherent differences, the overall consistency in the results highlights the value of these tools in informing public health strategies and enhancing infectious disease control measures.

Therefore, we advise that while these tools are valuable for general guidance and strategic planning, their use in specific evaluations should be supplemented with context-specific data and considerations. Future work should aim to refine these models, reduce uncertainties, and enhance their validation against real-world data to improve their applicability and reliability in diverse settings.

## CRediT authorship contribution statement

**Amar Aganovic:** Writing – original draft, Methodology, Investigation, Formal analysis, Conceptualization. **Giorgio Buonanno:** Writing – review & editing, Conceptualization. **Guangyu Cao:** Writing – review & editing, Conceptualization. **Christian Delmaar:** Writing – review & editing, Conceptualization. **Jarek Kurnitski:** Writing – review & editing, Conceptualization. **Alex Mikszewski:** Writing – review & editing, Conceptualization. **Lidia Morawska:** Writing – review & editing, Conceptualization. **Lucie C. Vermeulen:** Writing – review & editing, Conceptualization. **Pawel Wargocki:** Writing – review & editing, Conceptualization.

## Declaration of competing interest

The authors declare that they have no known competing financial interests or personal relationships that could have appeared to influence the work reported in this paper.
